# Vitamin C and Gallic Acid Ameliorate Motor Dysfunction, Cognitive Deficits, and Brain Oxidative Stress in a Valproic Acid‐Induced Model of Autism

**DOI:** 10.1002/brb3.70262

**Published:** 2025-02-05

**Authors:** Parnia Tarahomi, Mina Arab, Seyed Ali Seyedinia, Mehrnoush Rahmani, Ali Rashidy‐Pour, Abbas Ali Vafaei, Payman Raise‐Abdullahi

**Affiliations:** ^1^ Research Center of Physiology Semnan University of Medical Sciences Semnan Iran; ^2^ Department of Physiology, School of Medicine Semnan University of Medical Sciences Semnan Iran

**Keywords:** anxiety, autism, cognition, gallic acid, oxidative stress, valproic acid, vitamin C

## Abstract

**Purpose:**

Autism, a developmental‐neurodegenerative disorder, often manifests as social communication difficulties and has been correlated to oxidative stress in the brain. Vitamins C and gallic acid (GA) possess potent antioxidant properties, making them potential candidates for addressing autism‐related issues. This study examined the influence of vitamin C (Vit C) and GA on behavioral, motor, and cognitive performance, along with the assessment of brain oxidative markers, using an experimental model of autism.

**Method:**

Fourteen female rats were divided into saline and valproic acid (VPA) groups, and mating with mature male rats generated offspring. VPA (500 mg/kg) was injected intraperitoneally (i.p.) on gestational day (GD) 12.5. Male pups remained undisturbed for 29 days. On postnatal day (PND) 30, 48 male pups were randomly selected and administered daily injections of Vit C (30 mg/kg, i.p.) or GA (30 mg/kg, i.p.) for 4 weeks (PND 38–65). Behavioral assessments were conducted before and after treatment (PND 30–37 and 66–73). Animals were then anesthetized, and their brains were analyzed for oxidative stress markers.

**Finding:**

The prenatal VPA‐induced autism model increased nociceptive threshold, heightened anxiety‐like behaviors, impaired balance power, delayed spatial learning, elevated malondialdehyde, and decreased glutathione and catalase levels in the brains of the male offspring. Administration of Vit C and GA effectively mitigated these anomalies.

**Conclusions:**

Vit C and GA could potentially alleviate anxiety‐like behaviors, motor and cognitive deficits, and brain oxidative stress markers in a prenatal rat autism model. This underscores their viability as potential pharmacological interventions for treating autistic dysfunction.

## Graphical abstract

This graphical abstract illustrates the experimental design and findings of the study. Prenatal administration of valproic acid (VPA) at 500 mg/kg (i.p.) induced autism‐like behaviors in offspring, including increased pain tolerance, heightened anxiety‐like behaviors, motor dysfunction, and impaired spatial learning. Postnatal treatment with vitamin C (30 mg/kg, i.p.) and gallic acid (30 mg/kg, i.p.) improved these deficits by reducing pain sensitivity and anxiety‐like behaviors while enhancing motor function and spatial learning. The treatments also modulated oxidative stress markers in the brain, increasing catalase (CAT) and glutathione (GSH) levels while reducing malondialdehyde (MDA). These pre‐clinical findings highlight the therapeutic potential of vitamin C and gallic acid in mitigating behavioral and neurochemical abnormalities associated with autism.

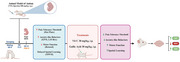

## 1 Introduction

Autism spectrum disorder (ASD), a developmental and neurodevelopmental disorder, is characterized by a triad of symptoms, including challenges in social interaction, verbal communication difficulties, and stereotyped behaviors. ASD is diagnosed in roughly 1 out of every 100 children worldwide (Zeidan et al. 2022). The average age at which autism is diagnosed varies widely, ranging from approximately 38 to 120 months (van ’t Hof et al. 2021). The etiology of autism involves both genetic and environmental factors. Individuals with ASD exhibit a range of pronounced movement issues, such as dyspraxia, ataxia, and hypersensitivity to specific stimuli. These symptoms manifest alongside altered pain sensitivity, visual and auditory processing disruptions, diverse gastrointestinal problems, cerebral edema, and hormonal imbalances (Restrepo, Enriquez, and Hansen 2022). Additionally, behavioral disturbances, like finger sucking, nail biting, repetitive motions, and stuttering, are common, leading to disrupted family interactions and detrimental impacts on learning, communication, and social functioning.

Presently, oxidative stress stands recognized as a pivotal factor contributing to the development and pathogenesis of ASD. Investigative studies into the connection between oxidative stress and autism have unveiled a disruption in the equilibrium between generating and neutralizing free radicals within ASD patients. This imbalance is mediated through distinct avenues: (1) perturbations within the mitochondrial electron transport complex, (2) peri‐, per‐, and postnatal risk factors like fetal distress, and (3) genetic factors encompassing gene polymorphisms associated with glutathione (GSH) metabolism, all culminating in the manifestation of oxidative stress conditions. This cascade subsequently contributes to the onset of autism by influencing brain tissue, the immune system, and the central nervous system's (CNS) mitochondria (Bjørklund et al. 2020).

Notably, investigations measuring lipid peroxidation, antioxidants, and antioxidant proteins such as ceruloplasmin in children diagnosed with ASD reveal elevated markers of lipid peroxidation alongside diminished levels of crucial antioxidant proteins like ceruloplasmin and transferrin. The notable correlation between reduced levels of these proteins and the regression of language skills in autistic children underscores the role antioxidants and oxidative stress play in this disorder's context (Chauhan and Chauhan 2006). Recent studies in this domain emphasize that utilizing diverse antioxidants harnessing their capacity to combat free radicals can reduce patient expenditures, enhance their quality of life, and ameliorate behavioral functions (Alasalvar and Bolling 2015; Fusco et al. 2007; Saadat et al. 2023; Yaribeygi et al. 2024).

Vitamin C (Vit C), known as ascorbic acid, holds paramount significance as a water‐soluble vitamin, pivotal in many physiological functions within the body (Naidu 2003). Its role as an antioxidant renders it a guardian against the deleterious effects of free radicals (Iqbal, Khan, and Khattak 2004). Within the brain and the CNS, ascorbate serves various functions. It actively counteracts oxygen and nitrogen free radicals generated during regular cellular metabolism and effectively neutralizes superoxide within neuronal tissue (14). Given its antioxidant properties, ascorbate finds application in treating neurodegenerative disorders like Alzheimer's, Parkinson's, and Huntington's diseases (Montine et al. 2002; Praticò 2002). Gallic acid (GA), denoted as 3, 4, 5‐trihydroxy benzoic acid, is an organic compound esteemed for its robust antioxidant characteristics and antimicrobial and anti‐cancer effects (Rahimi Asl et al. 2014). Its antioxidant prowess is attributed to the inhibition of tyrosine kinase activity and the augmentation of GSH peroxidase (GSH‐Px) enzyme levels. Moreover, it demonstrates pharmacological attributes encompassing antimicrobial effects and the induction of apoptosis in cancer cells (Locatelli et al. 2009; Rafiei, Bazyar, and Edalatmanesh 2016; Sarjit, Wang, and Dykes 2015). Notably, GA proves efficacious in alleviating an array of neurological disorders, including Alzheimer's (Rafiei, Bazyar, and Edalatmanesh 2016). This versatile organic substance is also utilized in the treatment of albuminuria and diabetic nephropathy. Remarkably, GA has been identified as the most potent active constituent within green tea leaves.

Owing to their inherent antioxidant properties, both Vit C and GA possess the potential to influence the development and severity of oxidative stress conditions. Consequently, these compounds hold promise in potentially impeding the progression of severe cerebral complications among individuals with autism by enhancing the functionality of the antioxidant defense system and counteracting reactive oxygen species (ROS). Notably, diminished levels of antioxidant proteins are closely linked to the deterioration of language skills in children with ASD. Consequently, it can be inferred that antioxidant therapies, such as GA, which augments GSH‐Px, could potentially ameliorate various other behavioral disorders exhibited by these patients.

Although antioxidant interventions show promise in neurodevelopmental disorders, there remains a critical gap in understanding their therapeutic potential, specifically in ASD. Although both Vit C and GA have demonstrated neuroprotective properties in various neurological conditions, their combined investigation in experimental models of autism remains unexplored. The valproic acid (VPA)‐induced autism model provides a well‐established platform to examine behavioral and biochemical alterations reminiscent of ASD. Notably, although previous studies have examined various antioxidant compounds in autism models, the specific effects of Vit C and GA—two compounds with distinct yet complementary antioxidant mechanisms—have not been investigated in the VPA model. Given Vit C's role in neural protection and GA's unique ability to enhance GSH‐Px activity, we hypothesized that these compounds could offer synergistic benefits in ameliorating autistic‐like symptoms. Therefore, this study aimed to investigate the effects of Vit C and GA administration on motor, cognitive, and behavioral deficits, as well as brain oxidative stress markers in the VPA‐induced autism model, potentially offering new insights into antioxidant‐based therapeutic strategies for ASD.

## 2 Materials and Methods

### 2.1 Animals

A total of 14 female Wistar rats (weighing 220–240 g) and 48 of their male offspring were used in this study. The pups were housed in groups of five per cage, maintained on a 12‐h light/dark cycle at a temperature of 22°C–24°C, and were provided with unlimited access to food and water. The study protocol received approval from the Ethical Review Board of Semnan University of Medical Sciences, Semnan, Iran (Approval No: IR.SEMUMS.REC.1400.106). All experimental procedures adhered to the guidelines set forth by the National Research Council Guide for the Care and Use of Laboratory Animals.

### 2.2 Induction of Autistic‐Like Behaviors

Female rats were randomly divided into two distinct groups. One group was designated as the sham (SH) group that received saline (SAL), intended for producing healthy offspring, whereas the other was referred to as the VPA group that received VPA, aimed at generating offspring displaying autistic‐like features. Subsequently, the female rats were randomly introduced to mature male rats for mating and offspring production. The initiation of pregnancy was determined by observing a vaginal plug, marking the first day of pregnancy. At gestational day 12.5 (GD 12.5), the VPA group received an intraperitoneal (i.p.) injection of VPA (500 mg/kg/2 mL) to induce autism‐like characteristics in the resultant offspring. In contrast, the SAL group received an equivalent volume of SAL (2 mL/kg) (Seyedinia et al. 2023).

### 2.3 Drugs

The Vit C (30 mg/kg/2 mL, i.p.) and GA (30 mg/kg/2 mL, i.p.), Sigma Co. USA, were dissolved in 0.9% SAL and injected daily (30 mg/kg/2 mL, i.p.). The SAL groups were administered 0.9% SAL equivalent to the vehicle (2 mL/kg).

### 2.4 Hot Plate

The hot plate test was conducted on postnatal days (PND) 30 and 66. For this test, the plate was heated to a temperature of 52°C. Each animal was placed on the plate, and the latency to respond to the heat by either licking its paws or attempting to jump out of the container was recorded in seconds. To ensure the safety of the animals and prevent tissue damage, a maximum cutoff time of 20 s was established (Seyedinia et al. 2023).

### 2.5 Rotarod Test

The rotarod test was administered on PND 31 and 67 to assess motor coordination and balance. Initially, the animals were placed on a horizontal bar to acclimate them to the apparatus. The rotarod was then set to rotate at a speed of 10 rpm to evaluate their balance. The duration each animal remained on the rotating bar was recorded, providing a measure of their motor coordination and balance capabilities (Seyedinia et al. 2023).

### 2.6 Elevated Plus Maze (EPM)

The EPM test was conducted on PNDs 32 and 68 to assess anxiety‐like behaviors. The EPM apparatus, constructed from wood, featured four arms arranged in a cross shape. Two of the arms were open, measuring 50 cm by 10 cm, whereas the other two arms were enclosed by dark walls, each measuring 40 cm in height, 50 cm in length, and 10 cm in width. The entire setup was elevated 50 cm above the ground. During the test, each animal was placed in the central area facing one of the open arms and allowed to explore the maze freely for 5 min. A number of entries into the open and closed arms, as well as the total time spent in each type of arm, were recorded. An entry into an arm was defined as having all four legs of the animal within that arm (Abdullahi et al. 2020).

### 2.7 Light–Dark Box (L/D Box)

The L/D Box test was conducted on PNDs 32 and 68 to assess anxiety‐like behaviors in rats. The apparatus consisted of a Plexiglass rectangular enclosure with dimensions of 61 cm in length, 20 cm in width, and 20 cm in height. It was divided into two distinct compartments: a light chamber (> 350 lx illumination intensity) and a dark chamber, each measuring 30 cm in length. A guillotine‐style door (7.5 × 7.5 cm^2^) separated the two chambers, allowing easy transfer between the compartments (Pereira, Gelfuso É, and Beleboni 2024).

The test was conducted in a controlled environment with consistent lighting and ambient noise levels to ensure uniformity across all sessions. Lighting in the test room was maintained at a constant level, and any extraneous noise was minimized to avoid influencing the animal's behavior. The test was performed during the light phase of the animals’ circadian cycle to avoid confounding results due to natural activity patterns. During the test, each subject was initially placed in the light chamber, facing the open end. The animal was then allowed to explore freely for a 5‐min duration, whereas its anxiety‐related behaviors were monitored. The following two key parameters were recorded: entrance latency (EL): The time the animal took to enter the dark chamber from the light chamber. Time spent in the light box (TLB): The total duration the animal remained in the light chamber, indicating its level of anxiety.

### 2.8 Morris Water Maze (MWM)

The MWM test was conducted on PND 33–37 and 69–73 to evaluate spatial learning and memory. The apparatus consisted of a circular metal tank with a diameter of 140 cm and a height of 55 cm, filled with water maintained at a temperature of 22°C–24°C and a depth of 25 cm. The pool was divided into four equal quadrants: northwest (NW), northeast (NE), southeast (SE), and southwest (SW). A transparent Plexiglas platform, 11 cm in diameter, was submerged about 2 cm below the water's surface, making it invisible to the animals. The platform remained in the same quadrant throughout the testing sessions to allow consistent spatial learning. To minimize spatial learning biases, the animals were introduced into the tank from varying starting points (NW, NE, SE, and SW) in a randomized sequence across trials (Hu et al. 2017).

The room housing the maze was equipped with distinct spatial cues, such as posters, shelves, and windows, which remained fixed throughout the study to aid navigation and ensure reliable spatial recognition. Environmental conditions were carefully controlled: lighting was uniform (20 lx illumination intensity), ambient noise was minimized, and testing occurred at the same time of day to avoid circadian rhythm effects. A high‐resolution camera, mounted 2 m above the tank, tracked the animals’ movements. These were analyzed using a computer‐based tracking system (EthoVision XT 7 software, Noldus, Wageningen, the Netherlands), allowing precise measurement of variables such as the latency to locate the platform, the distance swum, the time spent in each quadrant, and swimming speed. This standardized setup ensured consistent and accurate assessment of spatial learning and memory.

#### 2.8.1 Habituation

On PND 33, the rats were allowed to swim in the tank for 3 min with the platform removed to acclimate to the maze environment.

#### 2.8.2 Training

Between PND 33–36 and 69–72, the rats underwent training for four daily sessions over 4 consecutive days. The objective was for the rats to locate the platform in the center of one of the four quarters of the tank. In each training session, the rats were randomly introduced into the water from one of the four cardinal points of the tank (north, south, east, west). Subsequently, the rats had to navigate the water to locate and rest on the Plexiglas platform. Upon successfully reaching the platform, the rats were allowed a 30‐s stay. If a rat failed to find the platform within 60 s, it was gently guided to it by hand. Measurements included the time taken to find the platform and the total distance swum during each training session. Following the last training session, the animals were removed from the tank, dried with a towel, and returned to their respective cages.

#### 2.8.3 Learning Index (LI)

The LI is the percentage of EL reduction from Days 1 to 4. A higher LI signifies enhanced learning capabilities, serving as a benchmark for comparing the extent of learning across different groups. The LI was calculated with the following formula: [(escape latency in Day 1–escape latency in Day 4)/escape latency in Day 1] × 100.

#### 2.8.4 Recall Test

On PND 37 and 73, 1 day after the conclusion of the training sessions, the rats’ spatial memory was assessed. A 60‐s test was conducted during which the platform had been removed from the water. The measured parameters included the latency to cross the previous platform location, swimming speed, and time spent in the target zone.

### 2.9 Measurement of the Oxidative Stress Markers in the Brain

At the conclusion of the study, all rats were anesthetized using CO_2_, followed by decapitation. Their brains were carefully extracted for further analysis. A portion of each brain was rinsed in cold 0.9% SAL and stored at −70°C until homogenization using a Polytron PT 2100 homogenizer (KINEMATICA AG, Switzerland).

For malondialdehyde (MDA) assays, brain tissue was homogenized in a 1:10 weight‐to‐volume ratio with cold 1.15% KCl (Sigma‐Aldrich, USA). For GSH and catalase (CAT) measurements, homogenates were prepared by mixing 100 mg of tissue with 1 mL of phosphate buffer (50 mmol/L, pH 7.5) containing 1 mM EDTA (Sigma‐Aldrich, USA). The homogenates were then centrifuged at 20,000 × *g* for 10 min at 4°C, and the resulting supernatants were used for biochemical analyses. Total protein levels in the supernatants were determined using the Bradford method (Bio‐Rad, USA), with bovine serum albumin (BSA) as the standard.

### 2.10 Experimental Groups

On PND 30, control male offspring were randomly divided into four groups: SH‐SAL, SH‐Vit C, SH‐GA, and SH‐Vit C + GA, with seven animals in each group. The abbreviations stand for SH, SAL, Vit C, and GA. Similarly, autistic pups were divided into AUT‐SAL, AUT‐Vit C, AUT‐GA, and AUT‐Vit C + GA groups, with AUT indicating autistic.

Initial behavioral tests (pre‐treatment) were conducted from PND 30 to 37. Following these tests, the animals underwent 4 weeks of drug administration, starting on PND 38 and continuing until PND 65. The treatments included i.p. injections of Vit C (30 mg/kg), GA (30 mg/kg), a combination of both (Vit C + GA), or SAL (2 mL/kg).

**FIGURE 1:**
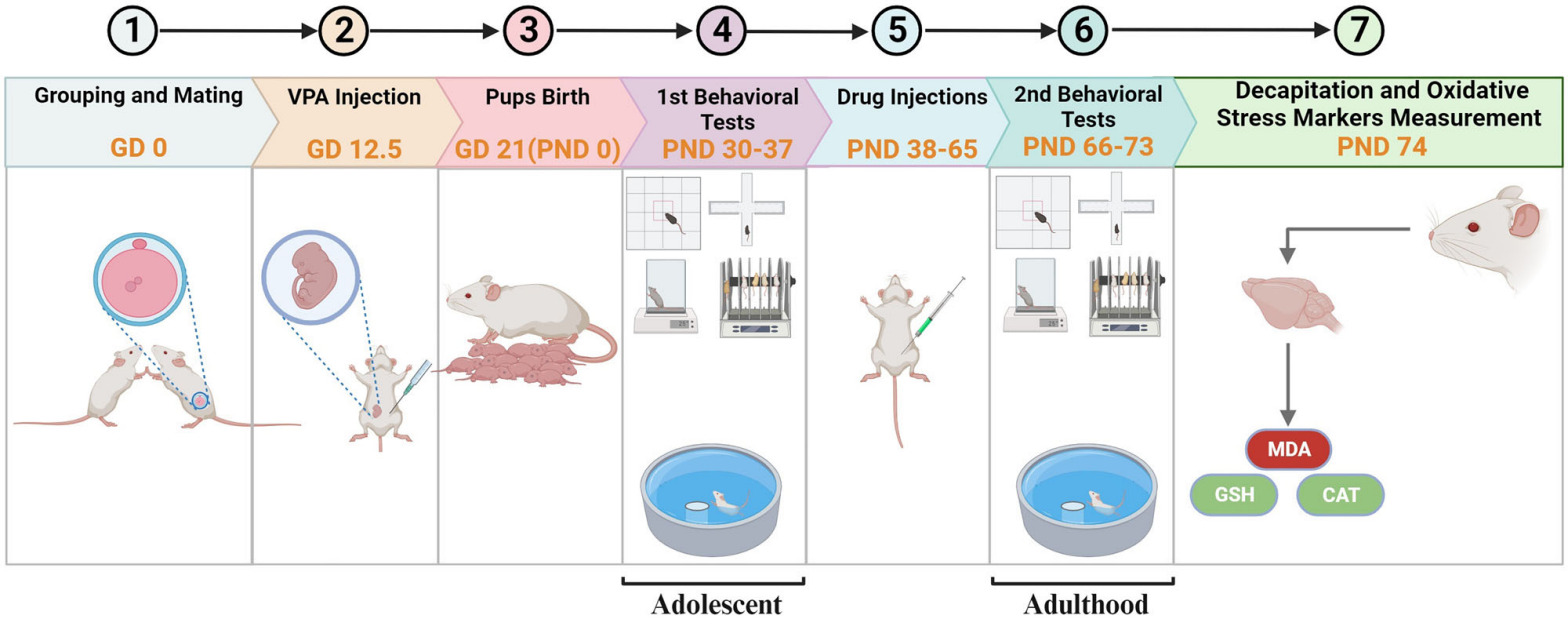
Timeline of behavioral tests and drug injections (see Section 2 for more detail). GD, gestation day; PND, postnatal day; VPA, valproic acid.

After the treatment period, a second round of behavioral tests (post‐treatment) was conducted from PND 66 to 73 to evaluate the effects on behavior and cognitive performance. Finally, the animals were sacrificed under deep anesthesia, and their brains were collected for the assessment of oxidative stress markers, including MDA, GSH, and CAT, using a commercial kit (Figure 1).

### 2.11 Statistical Analysis

The data were presented as mean ± SEM. The Shapiro–Wilk test was initially used to assess the normality of the data, and the homogeneity of variances was verified using Levene's test. Because the data met the assumption of normality and homogeneity of variances, two‐way or three‐way repeated‐measures ANOVA was performed, followed by Tukey's post hoc test for pairwise comparisons. The significance level was set at *p* < 0.05.

## 3 RESULTS

### 3.1 Hot Plate Test

The data from the nociceptive threshold (NT) measurement are presented in Figure 2A.

FIGURE 2The effects of vitamin C and gallic acid on VPA‐induced pain, motor deficits, and anxiety‐like behaviors in male offspring rats. (A) Hot plate test to evaluate nociceptive threshold; (B) rotarod test to evaluate balance times; (C and D) elevated plus maze and (E and F) light‐dark box, respectively, to evaluate anxiety‐like behaviors. Data are expressed as mean ± SEM. #*p* < 0.05 and ##*p* < 0.01 versus the SH‐SAL group. *p < 0.05 and **p < 0.01 versus the AUT‐SAL group. AUT: autistic; SAL: saline; SH: sham; Vit C, vitamin C.

**Figure:**
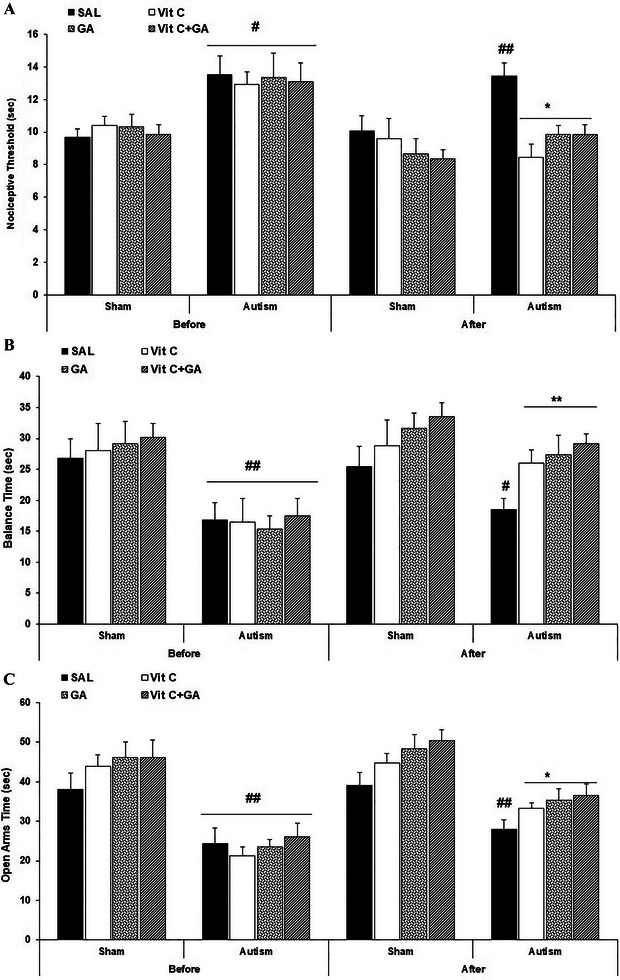

**Figure:**
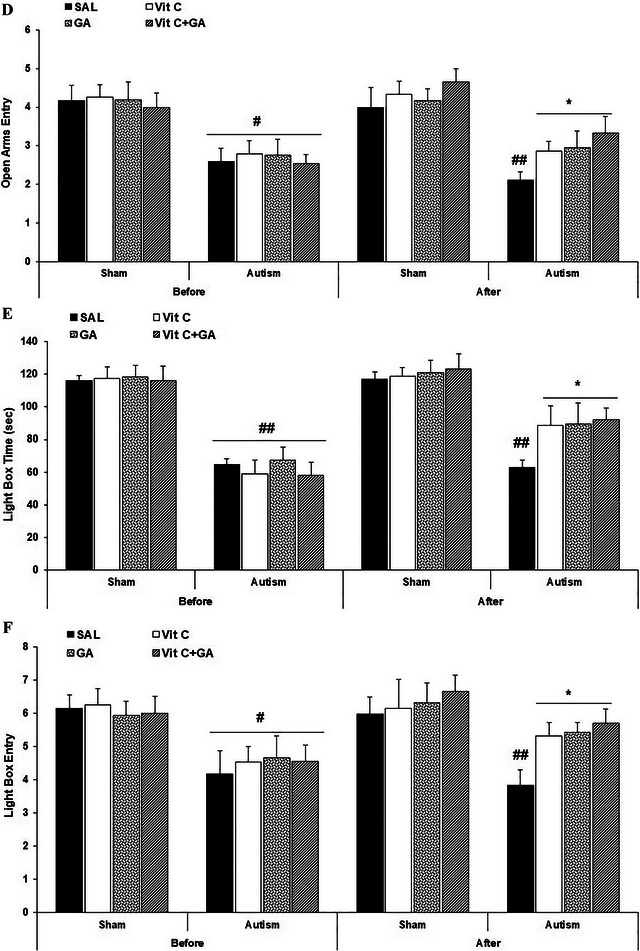

A two‐way repeated‐measures ANOVA [time (before or after treatment) × group (SH or autism)] on NT indicated significant main effects of time (*F*_1,10_ = 14.88, *p* = 0.003) and group (*F*_7,70_ = 4.69, *p* = 0.0002), and a significant interaction between time and group (*F*_7,70_ = 2.50, *p* = 0.05).

A Tukey post hoc analysis revealed that NT on PND 30 (before treatment) was significantly elevated in the AUT‐SAL, AUT‐Vit C, AUT‐GA, and AUT‐Vit C + GA groups compared with the SH‐SAL, SH‐Vit C, SH‐GA, and SH‐Vit C + GA groups, respectively (*p* < 0.05). Additionally, NT on PND 66 (after treatment) demonstrated an increase in the AUT‐SAL group compared with the SH‐SAL group (*p* < 0.05). However, a significant reduction in NT was observed in the AUT‐Vit C, AUT‐GA, and AUT‐Vit C + GA groups compared with the AUT‐SAL group (*p* < 0.01).

In summary, administering VPA to pregnant rats led to the development of autism‐like behaviors in their male offspring. Notably, the autistic groups exhibited a significantly heightened NT compared with the SH (healthy) groups. This observation underscores how the experimental autism model extended the duration of response to peripheral thermal pain. Interestingly, both individual and combined treatments of Vit C and GA displayed substantial improvements in autism‐like behaviors and the response to peripheral thermal pain when contrasted with the effects of SAL treatment.

### 3.2 Rotarod Test

The data from the Balance Times (BTs) measurement are presented in Figure 2B.

A two‐way repeated‐measures ANOVA on BT indicated significant main effects of time (*F*_1,10_ = 4.43, *p* = 0.05) and group (*F*_7,70_ = 4.24, *p* = 0.0006), but no significant interaction between time and group (*F*_7,70_ = 0.44, *p* = 0.86). The Tukey post hoc analysis revealed that the BT on PND 31 (before treatment) was reduced in the AUT‐SAL, AUT‐Vit C, AUT‐GA, and AUT‐Vit C + GA groups compared with the SH‐SAL, SH‐Vit C, SH‐GA, and SH‐Vit C + GA groups, respectively (*p* < 0.01). Furthermore, BT on PND 67 (after treatment) was reduced in the AUT‐SAL group compared with the SH‐SAL group (*p* < 0.05). However, BT was higher in the AUT‐Vit C, AUT‐GA, and AUT‐Vit C + GA groups compared with the AUT‐SAL group (*p* < 0.01).

In summary, the BT was notably reduced in the groups displaying autistic traits compared with the SH (healthy) groups. This finding signifies that the autism experimental model contributes to impaired balance and motor activity. The administration of Vit C and GA, either individually or in combination, significantly improved balance and motor activity compared with the effects of SAL treatment.

### 3.3 EPM Test

The data from the Open Arms Time (OAT) are presented in Figure 2C.

A two‐way repeated‐measures ANOVA on OAT indicated significant main effects of time (*F*_1,10_ = 13.03, *p* = 0.004) and group (*F*_7,70_ = 15.79, *p* < 0.0001), but no significant interaction between time and group (*F*_7,70_ = 0.79, *p* = 0.59). The Tukey post hoc analysis revealed that the OAT on PND 32 (before treatment) was decreased in the AUT‐SAL, AUT‐Vit C, AUT‐GA, and AUT‐Vit C + GA groups compared with the SH‐SAL, SH‐Vit C, SH‐GA, and SH‐Vit C + GA groups, respectively (*p* < 0.01). Furthermore, OAT on PND 68 (after treatment) was decreased in the AUT‐SAL group compared with the SH‐SAL group (*p* < 0.01). However, OAT was increased in the AUT‐Vit C, AUT‐GA, and AUT‐Vit C + GA groups compared with the AUT‐SAL group (*p* < 0.05).

The data from the Open Arms Entry (OAE) are presented in Figure 2C.

A two‐way repeated‐measures ANOVA on OAE indicated significant main effects of time (*F*_1,10_ = 12.01, *p* = 0.006) and group (*F*_7,70_ = 7.55, *p* < 0.0001), but no significant interaction between times and group (*F*_7,70_ = 1.36, *p* = 0.23). The Tukey post hoc analysis revealed that the OAE on PND 32 (before treatment) was reduced in the AUT‐SAL, AUT‐Vit C, AUT‐GA, and AUT‐Vit C + GA groups compared with the SH‐SAL, SH‐Vit C, SH‐GA, and SH‐Vit C + GA groups, respectively (*p* < 0.05). Furthermore, OAE on PND 68 (after treatment) was increased in the AUT‐SAL group compared with the SH‐SAL group (*p* < 0.01). However, OAE was raised in the AUT‐Vit C, AUT‐GA, and AUT‐Vit C + GA groups compared with the AUT‐SAL group (*p* < 0.05).

In summary, the time spent in the open arms and the frequency of entries into the open arms were significantly lower in the groups manifesting autistic characteristics compared with the SH (healthy) groups. These findings strongly indicate that this experimental model of autism increased anxiety‐like behaviors in rats. Remarkably, the application of Vit C and GA, both individually and in combination, significantly alleviated the anxiety‐like behaviors compared with the effects of the vehicle.

### 3.4 L/D Box Test

The data from the TLB are presented in Figure 2E.

A two‐way repeated‐measures ANOVA on TLB indicated significant main effects of time (*F*_1,10_ = 2.93, *p* = 0.05) and group (*F*_7,70_ = 16.78, *p* < 0.0001), but no significant interaction between time and group (*F*_7,70_ = 0.82, *p* = 0.56). The Tukey post hoc analysis revealed that the TLB on PND 32 (before treatment) was decreased in the AUT‐SAL, AUT‐Vit C, AUT‐GA, and AUT‐Vit C + GA groups compared with the SH‐SAL, SH‐Vit C, SH‐GA, and SH‐Vit C + GA groups, respectively (*p* < 0.01). Furthermore, TLB on PND 68 (after treatment) was decreased in the AUT‐SAL group compared with the SH‐SAL group (*p* < 0.01). However, TLB was higher in the AUT‐Vit C, AUT‐GA, and AUT‐Vit C + GA groups compared with the AUT‐SAL group (*p* < 0.05).

The data from the EL are presented in Figure 2F.

A two‐way repeated‐measures ANOVA on EL indicated significant main effects of time (*F*_1,10_ = 16.07, *p* = 0.002) and groups (*F*_7,70_ = 3.55, *p* = 0.002) and a significant interaction between times and groups (*F*_7,70_ = 2.04, *p* = 0.05). The Tukey post hoc analysis revealed that the EL on PND 32 (before treatment) was decreased in the AUT‐SAL, AUT‐Vit C, AUT‐GA, and AUT‐Vit C + GA groups compared with the SH‐SAL, SH‐Vit C, SH‐GA, and SH‐Vit C + GA groups, respectively (*p* < 0.05). Furthermore, EL on PND 68 (after treatment) was reduced in the AUT‐SAL group compared with the SH‐SAL group (*p* < 0.01). However, EL was higher in the AUT‐Vit C, AUT‐GA, and AUT‐Vit C + GA groups compared with the AUT‐SAL group (*p* < 0.05).

In summary, the TLB and the EL to the light box were reduced in the groups displaying autistic traits compared with the SH (healthy) groups. These findings strongly suggest that the experimental autism model led to an elevation in anxiety‐like behaviors. Importantly, administering Vit C and GA, either individually or in combination, significantly mitigated the anxiety‐like behaviors compared with the effects of SAL treatment.

### 3.5 MWM Test

**FIGURE 3:**
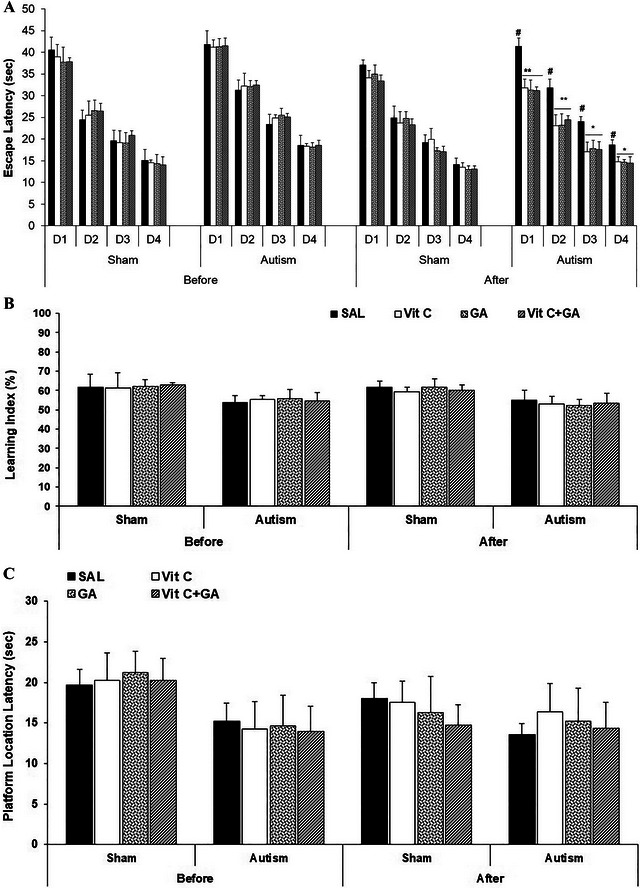
The effects of vitamin C and gallic acid on VPA‐induced spatial learning and memory deficits. (A) Escape latency; (B), learning index; (C), platform location latency (Prob Test). Data are expressed as mean ± SEM. #*p* < 0.05 versus the corresponding SH‐SAL groups. *p < 0.05 and **p < 0.01 versus the corresponding AUT‐SAL groups. AUT: autistic; SAL: saline; SH: sham; Vit C, vitamin C.

The data from the EL in the training phase are presented in Figure 3A.

A three‐way repeated‐measures ANOVA [group (SH or autism) × time (before or after treatment) × days (days of training)] on EL indicated no significant effect of group (*F*_1,20_ = 0.14, *p* = 0.90) but significant effects of time (*F*_1,20_ = 125.69, *p* < 0.0001) and days (*F*_3,60_ = 7.71, *p* = 0.002), significant interactions between group and time (*F*_1,20_ = 21.41, *p* = 0.0002), and groups and days (*F*_3,60_ = 4.54, *p* = 0.006), no significant interaction between time and days (*F*_3,60_ = 2.05, *p* = 0.11), and a significant interaction among group, time, and days (*F*_3,60_ = 6.48, *p* = 0.0007). The Tukey post hoc analysis indicated that the EL (both pre‐ and post‐treatment) decreased across all groups from Days 1 to 4 during the periods of PND 33–36 and 69–72 (*p* < 0.05; symbols are not shown for simplicity). On PND 69–72, EL was elevated in the AUT‐SAL group compared with the SH‐SAL group (*p* < 0.01). However, EL was reduced in the AUT‐Vit C, AUT‐GA, and AUT‐Vit C + GA groups compared with the AUT‐SAL group (*p* < 0.01 for Days 1 and 2, *p* < 0.05 for Days 3 and 4).

The data from the LI are presented in Figure 3B.

A two‐way repeated‐measures ANOVA on LI indicated no significant main effects of time (*F*_1,22_ = 0.69, *p* = 0.41) and group (*F*_3,66_ = 0.11, *p* = 0.95) and no significant interaction between time and group (*F*_3,66_ = 0.1, *p* = 0.95).

The data from the probe test (platform location latency; PLL) are presented in Figure 3C.

A two‐way repeated‐measures ANOVA on the PLL indicated no significant main effects of time (*F*_1,10_ = 0.11, *p* = 0.75) and groups (*F*_7,70_ = 1.03, *p* = 0.41) and no significant interaction between time and groups (*F*_7,70_ = 1.12, *p* = 0.37).

In summary, all animals showed decreased escape latencies from Days 1 to 4, indicating the learning. Autistic adult rats showed significantly longer escape latencies than healthy subjects, indicating delayed learning. Adolescent rats did not show this difference. This finding highlights that the experimental autism model caused delayed learning, specifically in adulthood. The separate administration of Vit C and GA, as well as their combined treatment, substantially improved spatial learning compared with SAL treatment in autistic adult rats. Interestingly, the homogenous LI indicated that although adult autistic rats initially demonstrated slower acquisition of the spatial learning task than healthy rats, they eventually learned the task. These findings were also approved by homogenous results of MWM memory (Prob Test).

### 3.6 Brain Oxidative Stress Markers

**FIGURE 4:**
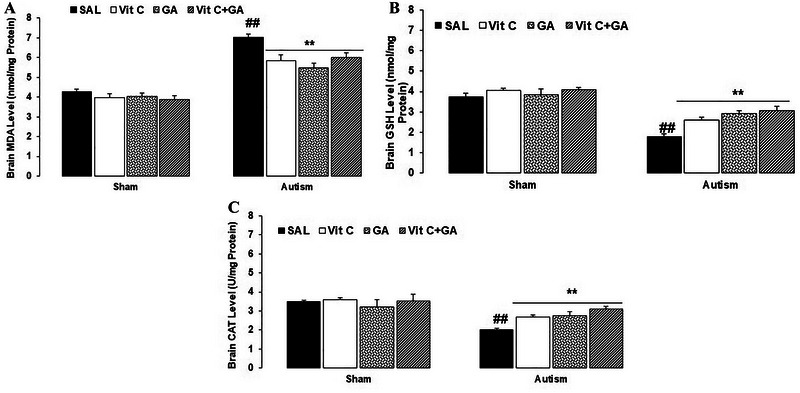
The effects of vitamin C and gallic acid on VPA‐induced brain oxidative stress markers in male offspring rats. (A) Malondialdehyde. (B) Glutathione. (3) Catalase. Data are expressed as mean ± SEM. ##*p* < 0.01 versus the SH‐SAL group. **p < 0.01 versus the AUT‐SAL group. AUT: autistic; SAL: saline; SH: sham; Vit C, vitamin C.

The data from oxidative stress markers are presented in Figure 4A–C.

A two‐way ANOVA [groups (SH or autistic) × treatments] on MDA levels revealed significant effects of groups (*F*_1,24_ = 218.37, *p* < 0.0001) and treatments (*F*_3,24_ = 8.65, *p* = 0.0005) and a significant interaction between groups and treatments (*F*_3,24_ = 4.25, *p* = 0.01). The Tukey post hoc analysis indicated that the MDA level on PND 74 (after treatment) was elevated in the AUT‐SAL compared with the SH‐SAL (*p* < 0.01). Moreover, MDA levels were reduced in the AUT‐Vit C, AUT‐GA, and AUT‐Vit C + GA compared with the AUT‐SAL (*p* < 0.01) (Figure 4A).

A two‐way ANOVA on GSH levels revealed significant effects of groups (*F*_1,24_ = 130.52, *p* < 0.0001) and treatments (*F*_3,24_ = 9.13, *p* = 0.0003) and a significant interaction between groups and treatments (*F*_3,24_ = 3.98, *p* = 0.02). The Tukey post hoc analysis indicated that the GSH level on PND 74 (after treatment) was reduced in the AUT‐SAL compared with the SH‐SAL (*p* < 0.01). Moreover, GSH levels were elevated in the AUT‐Vit C, AUT‐GA, and AUT‐Vit C + GA compared with the AUT‐SAL (*p* < 0.01) (Figure 4B).

A two‐way ANOVA on CAT levels revealed significant effects of groups (*F*_1,24_ = 28.12, *p* < 0.0001) and treatments (*F*_3,24_ = 2.43, *p* < 0.05) and a significant interaction between groups and treatments (*F*_3,24_ = 2.58, *p* < 0.05). The Tukey post hoc analysis indicated that the CAT level on PND 74 (after treatment) was reduced in the AUT‐SAL compared with the SH‐SAL (*p* < 0.01). Moreover, CAT levels were elevated in AUT‐Vit C, AUT‐GA, and AUT‐Vit C + GA compared with the AUT‐SAL (*p* < 0.01) (Figure 4C).

In summary, in the groups exhibiting autistic features, there was a notable rise in brain MDA levels, alongside a significant reduction in GSH and CAT levels compared to the SH (healthy) groups, suggesting that the experimental autism model triggered oxidative stress. Remarkably, the separate application of Vit C and GA and their combined treatment exhibited substantial improvements in brain oxidative stress markers, contrasting with the outcomes of SAL therapy.

## 4 DISCUSSION

The main important findings of this study are as follows: (1) The experimental prenatal VPA‐induced model of autism led to heightened anxiety‐like behaviors and NT, along with diminished balance power and delayed spatial learning, as well as increased MDA levels and decreased GSH and CAT levels in the brain of male offspring; (2) the individual administration of Vit C and GA, as well as their combined treatment, exhibited notable improvements in these outcomes (Table 1).

**TABLE 1:** Overview of behavioral evaluations and measurements of brain oxidative stress markers conducted in both healthy and valproic acid (VPA)‐induced autistic offspring rats before and after a 28‐day treatment regimen involving vitamin C and gallic acid.

Behavioral tests	Saline‐treated autistic rats	Vitamin C and gallic acid‐treated autistic rats
Hot plate	↑ Response to pain	↓ Response to pain
Rotarod test	↓ Balance time	↑ Balance time
Elevated plus maze	↓ Open Arms Time ↓ Open Arms Entry	↑ Open Arms Time ↑ Open Arms Entry
Light–dark box	↓ Time spent in the light box ↓ Light box entry	↑ Time spent in the light box ↑ Light box entry
Morris water maze	↑ Escaple latency –Platform location latency	↓ Escaple latency –Platform location latency

Brain oxidative stress markers	Saline‐treated autistic rats	Vitamin C and gallic acid‐treated autistic rats
Malondialdehyde	↑	↓
Glutathione	↓	↑
Catalase	↓	↑

The observed protective effects of Vit C and GA are likely attributable to their antioxidant properties. Meanwhile, studies investigating the interplay between oxidative stress and autism have revealed that in individuals with autism, the equilibrium between the production and neutralization of free radicals becomes disrupted through various mechanisms: (1) disorders in the mitochondrial electron transport complex; (2) risk factors during peri‐, per‐, and postnatal phases, such as complications during umbilical cord events, fetal distress, birth injuries, multiple births, premature delivery, low birth weight, exposure to smoking, heavy metals, and infections; and (3) genetic factors, including polymorphisms in genes associated with GSH metabolism. These pathways contribute to oxidative stress by interfering with oxidative phosphorylation of ATP, boosting ROS generation, and depleting the antioxidant defense system. Oxidative stress stands as a primary contributor to autism pathogenesis, precipitating: (1) mitochondrial dysfunction in CNS cells coupled with heightened lipid peroxidation, protein oxidation, and DNA damage; (2) inciting neuronal inflammation, initiating cerebral plaque formation, fostering aberrant neuronal growth, and releasing detrimental pro‐inflammatory cytokines; (3) triggering immune responses; and (4) inhibiting methionine synthase, which leads to epigenetic perturbations via diminished DNA methylation, ultimately disrupting brain development (Bjørklund et al. 2020). Collectively, these pathways underscore the profound role of oxidative stress in the initiation, progression, and complications associated with autism.

### 4.1 Effects of Vit C and GA Treatments on NT

The hot plate test revealed delayed nociceptive responses in the autistic rats. Subsequent GA and Vit C administration to the experimental groups demonstrated that the time required to respond to pain approached that of healthy rats. However, following treatment, the NT decreased. Similarly, Al‐Amin et al. explored the impact of astaxanthin, an antioxidant, on VPA‐induced autistic rats, aligning with our findings (Al‐Amin et al. 2015). They reported prolonged nociceptive response duration in autistic rats, which decreased after astaxanthin treatment. This study implies that astaxanthin might alleviate disrupted behaviors in the autism animal model through its antioxidant properties.

To rationalize this outcome, it can be postulated that brain neurons undergo damage during embryonic development due to oxidative stress, particularly via pathways like mitochondrial dysfunction. In addition to genetic factors, the brain secretes endogenous opioids to mitigate pain, thereby inducing a sense of euphoria. As the nervous system develops, the persistent presence of opioids alongside new neuron formation during embryogenesis leads neurons to adapt to opioid properties within the tissue environment (Nader et al. 2004; Pellissier et al. 2018). Consequently, abnormal signaling mechanisms form within nerve cells, disrupting feedback loops (Gidaya et al. 2016) and elevating the NT. Vit C and GA likely curtailed tissue damage, suppressed brain opioid release, restored feedback mechanisms, and ultimately lowered the pain threshold in the autistic model. These effects were potentially achieved by bolstering the antioxidant defense system and attenuating the impact of oxidative stress on brain tissues.

### 4.2 The Effects of Vit C and GA Treatments on Anxiety‐Like Behaviors

This investigation unveiled that the VPA‐induced autism model heightened anxiety‐like behaviors in the offspring, a trend markedly reversed by Vit C and GA treatment. Bhandari et al. explored the antioxidative potential of resveratrol in mitigating neuronal inflammation and neurobehavioral deficits linked to ASDs in rats. In‐line with our findings, their study concluded that resveratrol, by quelling oxidative/nitrosative stress and mitigating mitochondrial dysfunction, enhances autistic behaviors, including alleviating anxiety‐like behaviors (Bhandari and Kuhad 2017). To elucidate this outcome, it is worth noting that the brain contains a substantial proportion of oxidizable polyunsaturated fatty acids and redox‐active metals like iron and copper, rendering it susceptible to oxidative stress. Given oxidative stress's role in autism pathogenesis, one of the regions particularly vulnerable to oxidative damage is the amygdala, a region implicated in emotional expression and social impairments often seen in autistic individuals (Liu et al. 2022).

Research findings underscore the impact of oxidative stress and its resultant inflammation on amygdala morphology and neurotransmitter dysregulation, particularly glutamate, within autism models. Elevated glutamate activity has been associated with heightened neurotic behaviors (Carpita, Muti, and Dell'Osso 2018; Pardo and Eberhart 2007). Anxiety prompts the release of glucocorticoids and ROS due to escalated metabolic rates, thereby exacerbating brain oxidative stress (Uljarevic, Nuske, and Vivanti 2016). Vit C's antioxidant properties and its potential to modulate oxidative stress likely influence brain morphological and functional alterations. By counteracting the exacerbating effects of oxidative stress on the amygdala, Vit C possibly mitigates anxiety‐like behaviors in these rat models. In humans, low Vit C levels have been linked to anxiety, stress, depression, and fatigue, with its impact on mood attributed to mechanisms such as transporter regulation, cortisol activity reduction, prevention of stress‐induced oxidative damage, and reinforcement of brain antioxidant defenses.

Regarding GA, emerging evidence suggests that individuals with autism experience ROS accumulation (Andrade 2016) and disruption of antioxidant systems, including reduced levels of antioxidant enzymes like superoxide dismutase (SOD) and GSH‐Px, and increased MDA levels, which are associated with anxiety (Vorhees et al. 1994). GA likely ameliorated anxiety‐like behaviors in rats by inhibiting tyrosine kinase activity, augmenting GSH‐Px enzyme levels, and leveraging its antioxidant properties to fortify the rat's antioxidant defense system.

### 4.3 The Effects of Vit C and GA Treatments on Motor Balance

The current study unveiled disruptions in rats’ balance performance as evidenced by the rotarod test. However, intervention with GA and Vit C significantly ameliorated this impairment. In congruence with these findings, Morakotsriwan et al. (2016) explored the impact of antioxidant‐rich purple rice extract and silkworm supplementation on behavioral performance and histopathological alterations in the cerebellum of VPA‐induced autistic rats. Aligning with our study, their research indicated that rats receiving purple rice extract and silkworm exhibited improved balance performance in the rotarod test and other evaluations. Moreover, Purkinje cell loss and oxidative stress were reduced within the rats’ cerebellum, leading to decreased autistic‐like behaviors (Morakotsriwan et al. 2016). It can be postulated that oxidative stress during brain development manifests diverse effects on autism, contributing to GSH disruption and enhanced mitochondrial ROS production, collectively contributing to developmental disturbances across various brain regions, notably the cerebellum.

GSH exists in two primary forms: (1) thiol‐reduced (GSH) and (2) disulfide‐oxidized (GSSG). The GSH/GSSG balance, represented by the GSH/GSSG ratio, is a marker for oxidative stress. Neuroimaging and postmortem neuropathological investigations have highlighted the cerebellum as a prominent site where this ratio undergoes significant alterations in autistic patients, signifying escalated oxidative stress within this region (Becker and Stoodley 2013; Chauhan, Audhya, and Chauhan 2012). This oxidative stress contributes to the diminishment in both size and density of Purkinje cells, eventually culminating in cell loss. This cell loss predominantly manifests in the vermis and the cerebellar hemispheres.

Furthermore, effects, such as reduction in granular cells, atrophy of cerebellar nuclei, bilateral cortex reduction, and cerebellar enlargement, have also been observed (Becker and Stoodley 2013; Fatemi et al. 2012). Ultimately, the presence of antioxidants, such as Vit C and GA, likely mitigates the impact of oxidative stress on Purkinje cells and the cerebellum by fortifying the antioxidant defense and bolstering key antioxidant proteins like GSH. By ameliorating oxidative stress conditions, these interventions potentially forestall further neuronal damage and inflammation, consequently suppressing immune responses and enhancing balance conditions.

### 4.4 The Effects of Vit C and GA Treatments on Spatial Learning and Memory

The results of the MWM test unveiled that the experimental autism model significantly prolonged the latency of finding the hidden platform during the acquisition training phase in adult but not adolescent rats, indicating delayed spatial learning capabilities in adulthood. However, the administration of Vit C and GA markedly shortened the EL, underscoring the beneficial effects of these antioxidants on cognitive processes. These findings align with research by Adebiyi et al. (2022), which demonstrated that GA and Vit C administration alleviated cadmium chloride‐induced cognitive deficits, including enhancing performance in the MWM test.

Our results demonstrated that spatial learning eventually occurred in all experimental groups during the acquisition training phase of the MWM. However, compared with the control group, adult autistic rats exhibited significantly prolonged escape latencies when locating the hidden platform. Notably, the prenatal VPA model of autism delayed but did not wholly impair spatial learning abilities. The increased time taken by autistic rats to find the platform underscores the speed of learning deficits induced by developmental exposure to VPA. This finding has two key implications. First, the learning delay was specific to adulthood, as adolescent autistic rats did not show impaired spatial learning compared to healthy controls. Second, even with slower acquisition, the autistic adult rats were eventually able to learn the task, indicating their spatial learning capacity was intact but delayed. Importantly, this learning delay did not reflect memory impairment, as spatial memory performance was equivalent between autistic and healthy adult rats once the task was learned. Thus, the core deficit observed was in the speed of learning acquisition, not memory per se.

In addition, these findings have important implications for teaching individuals with autism. The slower acquisition of new spatial learning in adult autistic rats suggests that people with autism may initially struggle when learning new skills. However, it is critical to note that with enough time and proper instruction, the autistic rats eventually learned the task at hand. This indicates that the learning capacity of autistic individuals is intact, even if the learning rate is delayed. With appropriate support and teaching strategies tailored to their needs, people with autism can likely achieve learning outcomes comparable to neurotypical individuals. However, it is important that educators not mistake initial slower learning for an inability to learn; instead, they should persist and find suitable techniques to facilitate skill acquisition in autism.

We also found that autistic rats who received Vit C or GA treatment displayed markedly shorter escape latencies in finding the hidden platform during acquisition training. This demonstrates that administering these antioxidants significantly improved spatial learning capabilities in the VPA‐induced model of autism. The reduced time taken to locate the platform highlights the ameliorating effects of Vit C and GA on learning deficits associated with developmental VPA exposure.

The cognitive‐enhancing effects of Vit C and GA likely stem from their capacity to combat oxidative stress and reinstate GSH, CAT, and other endogenous antioxidant mechanisms within the hippocampus and associated limbic structures (Roidoung, Dolan, and Siddiq 2016). Oxidative stress is known to precipitate cognitive decline by damaging neuronal membranes, promoting tau hyperphosphorylation, disrupting synaptic plasticity, and triggering neuroinflammation. Additionally, GSH depletion within the hippocampus has been associated with impaired spatial learning and memory (Dean et al. 2009). Replenishing GSH and bolstering enzymatic antioxidants, like CAT, Vit C, and GA, potentially preserved neuronal integrity and function, consequently enhancing cognitive capabilities in the VPA‐induced rat model of autism. Furthermore, emerging evidence indicates that GA directly stimulates the brain‐derived neurotrophic factor (BDNF) pathway within the hippocampus (Zhu et al. 2019), which plays a pivotal role in learning, memory, and synaptic plasticity (Raise‐Abdullahi et al. 2023).

Hansen et al. have shown that Vit C deficiency leads to spatial learning and memory impairment (Hansen et al. 2018). Vit C has been shown to promote hippocampal neurogenesis and mitigate age‐related changes in the brain, resulting in enhanced learning and memory (Nam et al. 2019). The antioxidant properties of Vit C facilitate the growth and development of new neurons within the hippocampus. Vit C also protects against oxidative damage that can impair cognitive function. By stimulating neurogenesis and preserving neuronal integrity in the hippocampus, Vit C has demonstrated the ability to improve performance on learning and memory tasks that involve this brain region. Antioxidant treatment with Vit C and GA likely elicited protective effects against VPA‐induced spatial memory deficits through these mechanisms.

Eventually, the current study demonstrates the beneficial effects of Vit C and GA in ameliorating autism‐like behaviors and oxidative stress markers in a VPA‐induced model of autism in rats. Although these findings are promising, it is essential to contextualize the results by comparing them to other potential therapeutic approaches explored in autism research.

Several antioxidant compounds, such as *N*‐acetylcysteine (NAC) (Schiavi et al. 2022), polyphenolic compounds (Malaguarnera, Khan, and Cauli 2020; Seyedinia et al. 2023), *Ginkgo biloba* (Bahmani et al. 2016), a popular herbal supplement with an old history of administration in neuropsychological disorders (Alizadeh et al. 2024), and omega‐3 fatty acids (Veselinović et al. 2021), have been studied for their potential to mitigate oxidative stress and behavioral deficits in autism. NAC, for instance, is a precursor to GSH, a key endogenous antioxidant, and has shown efficacy in reducing irritability and repetitive behaviors in both animal models and clinical trials (Schiavi et al. 2022). Unlike Vit C and GA, which primarily act as direct free radical scavengers, NAC enhances the body's natural antioxidant defense systems. Although our findings highlight the ability of Vit C and GA to improve oxidative stress markers, further studies comparing their efficacy with NAC could provide a deeper understanding of their relative advantages. Moreover, omega‐3 fatty acids, widely known for their neuroprotective properties, have also been shown to improve behavioral symptoms in autistic children (Veselinović et al. 2021). Their effects are largely attributed to their role in modulating neuroinflammation and maintaining neuronal membrane integrity. Although omega‐3 fatty acids act on different mechanisms compared to Vit C and GA, combining these treatments may offer synergistic benefits, simultaneously addressing both oxidative stress and neuroinflammatory pathways. Similarly, resveratrol and crocin, polyphenolic compounds, have been reported to reduce neuroinflammation and oxidative stress in autism models (Malaguarnera et al. 2020; Seyedinia et al. 2023). Crocin's anti‐inflammatory properties, alongside its ability to modulate mitochondrial dysfunction, position it as a multifaceted therapeutic agent. In comparison, Vit C and GA primarily address oxidative damage, underscoring the need to explore combinatory therapies targeting multiple pathological pathways in autism.

Although our study focuses on the effects of Vit C and GA, it is evident that their therapeutic potential needs to be evaluated against or alongside other approaches to establish their relative efficacy. Future studies should aim to compare these antioxidants directly with other established or emerging treatments, both individually and in combination, to develop comprehensive therapeutic strategies for managing ASDs.

## 5 CONCLUSION

The prenatal VPA‐induced model of autism resulted in heightened NT, increased anxiety‐like behaviors, compromised balance, delayed spatial learning, elevated malondialdehyde levels, and reduced GSH and CAT levels in the brains of male offspring. These findings indicate that autistic offspring exhibit delays in spatial learning abilities, mirroring the initial struggles autistic individuals face when acquiring new skills. However, given sufficient time and appropriate guidance, the autistic rats were able to overcome these challenges and master the task. The administration of Vit C and GA effectively mitigated these impairments, suggesting their potential as pharmacological interventions for addressing the complexities of autism‐related dysfunction. However, it is important to recognize that results observed in animal models do not always translate directly to humans. Therefore, although these findings provide promising insights, further research, including clinical validation, is essential to confirm the safety and efficacy of these treatments in human populations.

## Author Contributions

**Parnia Tarahomi**: investigation. **Mina Arab**: investigation. **Seyed Ali Seyedinia**: investigation. **Mehrnoush Rahmani**: investigation. **Ali Rashidy‐Pour**: conceptualization, methodology. **Abbas Ali Vafaei**: conceptualization, methodology, investigation, supervision, writing–original draft, writing–review and editing. **Payman Raise‐Abdullahi**: writing–review and editing, writing–original draft, formal analysis.

## Ethics Statement

The Ethical Review Board of Semnan University of Medical Sciences, Semnan, Iran, approved the experimental protocol. All experiments were completed in agreement with the National Institutes of Health Guide for the Care and Use of Laboratory Animals. (NO: IR.SEMUMS.REC.1400.106).

## Conflicts of Interest

The authors declare no conflicts of interest.

### Peer Review

The peer review history for this article is available at https://publons.com/publon/10.1002/brb3.70262.

## Data Availability

The data that support the findings of this study are available from the corresponding author upon reasonable request.
